# Correction: Bismuthene nanosheets produced by ionic liquid assisted grinding exfoliation and their use for oxygen reduction reaction

**DOI:** 10.1039/d1ra90001c

**Published:** 2021-02-04

**Authors:** Manila Ozhukil Valappil, Abhijit Ganguly, John Benson, Vijayamohanan K. Pillai, Subbiah Alwarappan, Pagona Papakonstantinou

**Affiliations:** Engineering Research Institute, School of Engineering, Ulster University Newtownabbey BT37 0QB UK p.papakonstantinou@ulster.ac.uk; Electrodics and Electrocatalysis Division, CSIR-Central Electrochemical Research Institute Karaikudi Tamil Nadu 630003 India; 2-DTech Ltd, Core Technology Facility 46 Grafton St Manchester M13 9NT UK; Department of Chemistry, Indian Institute of Science Education and Research Tirupati India

## Abstract

Correction for ‘Bismuthene nanosheets produced by ionic liquid assisted grinding exfoliation and their use for oxygen reduction reaction’ by Manila Ozhukil Valappil *et al.*, *RSC Adv.*, 2020, **10**, 43585–43591, DOI: 10.1039/D0RA09763B.

The authors regret that Pagona Papakonstantinou’s email address was shown incorrectly in the original article. The corrected email address is as shown above.

The Royal Society of Chemistry apologises for these errors and any consequent inconvenience to authors and readers.

## Supplementary Material

